# Chloroplast protein StFC-II was manipulated by a *Phytophthora* effector to enhance host susceptibility

**DOI:** 10.1093/hr/uhae149

**Published:** 2024-05-28

**Authors:** Meng Xu, Xinyuan Sun, Xinya Wu, Yetong Qi, Hongjun Li, Jiahui Nie, Zhu Yang, Zhendong Tian

**Affiliations:** National Key Laboratory for Germplasm Innovation & Utilization of Horticultural Crops, Huazhong Agricultural University (HZAU), Wuhan 430070, China; Key Laboratory of Potato Biology and Biotechnology (HZAU), Ministry of Agriculture and Rural Affairs, Wuhan 430070, China; Potato Engineering and Technology Research Center of Hubei Province (HZAU), Wuhan 430070, China; National Key Laboratory for Germplasm Innovation & Utilization of Horticultural Crops, Huazhong Agricultural University (HZAU), Wuhan 430070, China; Key Laboratory of Potato Biology and Biotechnology (HZAU), Ministry of Agriculture and Rural Affairs, Wuhan 430070, China; Potato Engineering and Technology Research Center of Hubei Province (HZAU), Wuhan 430070, China; National Key Laboratory for Germplasm Innovation & Utilization of Horticultural Crops, Huazhong Agricultural University (HZAU), Wuhan 430070, China; Key Laboratory of Potato Biology and Biotechnology (HZAU), Ministry of Agriculture and Rural Affairs, Wuhan 430070, China; Potato Engineering and Technology Research Center of Hubei Province (HZAU), Wuhan 430070, China; National Key Laboratory for Germplasm Innovation & Utilization of Horticultural Crops, Huazhong Agricultural University (HZAU), Wuhan 430070, China; Key Laboratory of Potato Biology and Biotechnology (HZAU), Ministry of Agriculture and Rural Affairs, Wuhan 430070, China; Potato Engineering and Technology Research Center of Hubei Province (HZAU), Wuhan 430070, China; National Key Laboratory for Germplasm Innovation & Utilization of Horticultural Crops, Huazhong Agricultural University (HZAU), Wuhan 430070, China; Key Laboratory of Potato Biology and Biotechnology (HZAU), Ministry of Agriculture and Rural Affairs, Wuhan 430070, China; Potato Engineering and Technology Research Center of Hubei Province (HZAU), Wuhan 430070, China; National Key Laboratory for Germplasm Innovation & Utilization of Horticultural Crops, Huazhong Agricultural University (HZAU), Wuhan 430070, China; Key Laboratory of Potato Biology and Biotechnology (HZAU), Ministry of Agriculture and Rural Affairs, Wuhan 430070, China; Potato Engineering and Technology Research Center of Hubei Province (HZAU), Wuhan 430070, China; National Key Laboratory for Germplasm Innovation & Utilization of Horticultural Crops, Huazhong Agricultural University (HZAU), Wuhan 430070, China; Key Laboratory of Potato Biology and Biotechnology (HZAU), Ministry of Agriculture and Rural Affairs, Wuhan 430070, China; Potato Engineering and Technology Research Center of Hubei Province (HZAU), Wuhan 430070, China; National Key Laboratory for Germplasm Innovation & Utilization of Horticultural Crops, Huazhong Agricultural University (HZAU), Wuhan 430070, China; Hubei Hongshan Laboratory (HZAU), Wuhan 430070, China; Key Laboratory of Potato Biology and Biotechnology (HZAU), Ministry of Agriculture and Rural Affairs, Wuhan 430070, China; Potato Engineering and Technology Research Center of Hubei Province (HZAU), Wuhan 430070, China

## Abstract

Oomycete secretes a range of RxLR effectors into host cells to manipulate plant immunity by targeting proteins from several organelles. In this study, we report that chloroplast protein StFC-II is hijacked by a pathogen effector to enhance susceptibility. *Phytophthora infestans* RxLR effector *Pi22922* is activated during the early stages of *P. infestans* colonization. Stable overexpression of *Pi22922* in plants suppresses flg22-triggered reactive oxygen species (ROS) burst and enhances leaf colonization by *P. infestans*. A potato ferrochelatase 2 (FC-II, a nuclear-encoded chloroplast-targeted protein), a key enzyme for heme biosynthesis in chloroplast, was identified as a target of Pi22922 in the cytoplasm. The pathogenicity of Pi22922 in plants is partially dependent on FC-II. Overexpression of *StFC-II* decreases resistance of potato and *Nicotiana benthamiana* against *P. infestans*, and silencing of *NbFC-II* in *N. benthamiana* reduces *P. infestans* colonization*.* Overexpression of *StFC-II* increases heme content and reduces chlorophyll content and photosynthetic efficiency in potato leaves. Moreover, ROS accumulation both in chloroplast and cytoplasm is attenuated and defense-related genes are down-regulated in *StFC-II* overexpression transgenic potato and *N. benthamiana* leaves. Pi22922 inhibits E3 ubiquitin ligase StCHIP-mediated StFC-II degradation in the cytoplasm and promotes its accumulation in chloroplasts. In summary, this study characterizes a new mechanism that an oomycete RxLR effector suppresses host defenses by promoting StFC-II accumulation in chloroplasts, thereby compromising the host immunity and promoting susceptibility.

## Introduction

A two-layer innate immune system was built up in plants to fight pathogens during the long-term evolution process. Conserved pathogen-associated molecular patterns (PAMPs) are recognized by pattern recognition receptors (PRRs) to trigger PAMP-triggered immunity (PTI). Effector-triggered immunity (ETI) is caused by plant nucleotide-binding leucine-rich repeat (NLR) proteins that either directly or indirectly recognize the specific effector [1, 2]. PTI and ETI share many similar downstream responses [3–7]. A more comprehensive model of the plant immune system, consisting of three layers, multiple recognition, signal-integration, and diverse defense-action is proposed [8].

During the microbial pathogen infection processes, pathogens excrete a series of effectors to impair host immune responses and promote infection. Depending on their main site of action, effectors are divided into apoplastic and cytoplasmic effectors [2]. Cytoplasmic effectors are translocated inside the plant cell with multiple subcellular locations, in contrast to the apoplast-located effectors [9, 10]. Oomycete RxLR effector, which contains a conserved Arg-any amino acid-Leu-Arg (RxLR) motif, is a major class of identified cytoplasmic effectors that plays key roles in the arms race between plants and pathogens [10–12]. Diverse host processes, including transcription, translation, post-translational modifications, and intracellular trafficking etc. were manipulated by RxLR effectors [13–15, 55]. RxLR effectors regulate the activity of certain host enzymes. *Phytophthora infestans* Avr3a inhibits the activity of plant E3 ligase CMPG1 (a homolog, highly related to *Petroselinum crispum* CMPG1, is derived from the first four fully conserved residues: Cys, Met, Pro and Gly) [16, 17]. Potato positive immune regulators mitogen-activated protein kinase StMAP3Kɛ and StMAP3Kβ2 are targeted by effectors PexRD2 and Pi22926, respectively, to suppress cell death triggered by Cf4/Avr4 [18, 19]. Three catalytic isoforms of host protein phosphatase 1 (PP1c) are targeted by effector Pi04314 to form holoenzymes for promoting *P. infestans* colonization [20]. Some RxLR effectors affect the stability of plant proteins. For example, putative potato K-homology (KH) RNA-binding protein StKRBP1 is stabilized by effector Pi04089 to enhance infection [21]. StMKK1, a potato MAPK cascade protein, is targeted and stabilized by Pi20303 and Pi20300 to suppress plant PTI response [22]. Pi06432 stabilizes potato ubiquitin-like domain-containing protein StUDP to inhibit proteasome activity and host immunity [56]. The stability of desumoylating isopeptidase DeSI2 is disrupted by RxLR effector AVR8, resulting in suppression of host immunity [23]. Effectors also alter the subcellular localization of host targets. AVRblb2 inhibits the secretion of cysteine protease C14 into the apoplast, thus enhancing plant susceptibility [24]. *Phytophthora* Pi03192 disturbs the re-localization of NAC transcription factor NTP1 and NTP2 from the ER to the nucleus triggered by culture filtrate (CF) to suppress host defenses [25]. In addition, some effectors affect the complex formation of host proteins. Pi22798 promotes StKNOX3, a potato Knotted1-like homebox transcription factor, forming the homodimerization to enhance pathogenicity [26]. These examples demonstrate that RxLR effectors regulate plant immunity in multiple ways.

The chloroplast plays a key role in photosynthesis and plant immunity, which makes it a prime target to be attacked by pathogen virulence factors [27]. Chloroplast is a major generator of defense-related signaling molecules or their precursors such as salicylic acid (SA), abscisic acid (ABA) precursor, ethylene (ET) precursor, and jasmonates (JAs) precursor. Reactive oxygen species (ROS) burst and^**.**^NO accumulation require photosynthesis to provide carbon skeleton, energy and NADPH [28–30]. Pathogen effectors interact with various nuclear-coded chloroplast proteins to modulate chloroplast-mediated immunity [31, 32]. Some effectors interact with nuclear-coded chloroplast proteins in cytoplasm. For example, stripe rust effectors Pst_4 and Pst_5 weaken plant defenses by preventing TaISP translocation into chloroplasts, thereby reducing host ROS accumulation and promoting fungal infection [33]. *P. infestans* effector AVRvnt1 binds the full-length chloroplast-targeted GLYK isoform, activating Rpi-vnt1.1 for immunity. In the dark, truncated GLYK isoform (lacking the intact chloroplast transit peptide) is not targeted by AVRvnt1, resulting in attenuated Rpi-vnt1.1–mediated resistance. In the absence of Rpi-vnt1.1, AVRvnt1 intercepts GLYK’s trafficking to chloroplasts [34]. In addition, some effectors are secreted into chloroplasts to suppress chloroplast-mediated immunity by targeting the chloroplast proteins. HopI1 is one of the first identified bacterial chloroplast-localized effectors that alters thylakoid structure and reduces SA accumulation [35]. HopN1 from *Pseudomonas syringae* targets and degrades PsbQ, a component of photosystem II (PSII), thereby reducing oxygen production, electron transport, and suppressing chloroplast ROS (cROS) accumulation [36]. *Ralstonia solanacearum* chloroplast-localized effector RipAL induces JA production to suppress SA-mediated defense responses in plants [37]. Wheat stripe rust fungus effector PSTG_12806 targets the chloroplast and interacts with TaISP protein (a putative component of the cytochrome b6-f complex) to disturb chloroplast function [38]. *Plasmopara viticola* effector RxLR31154 targets and stabilizes PsbP (oxygen-evolving enhancer subunit) in grapevine chloroplasts, thereby inhibiting ROS production and leading to susceptibility [39].

In plant plastids, heme biosynthesis shares the same upstream pathway with chlorophyll biosynthesis until protoporphyrin IX is formed. Ferrochelatase (FC) is a terminal enzyme in the heme synthesis process, which catalyzes the incorporation of ferrous ions to the protoporphyrin IX ring to form heme [40, 41]. In land plants, there are two isoforms of FC: FC-I and FC-II [42]. *Arabidopsis* AtFC-II is imported solely into chloroplasts, whereas AtFC-I is transported to both chloroplasts and mitochondria [42, 43]. *FC-II* is predominantly expressed in photosynthetic tissues and light-induced, whereas *FC-I* is expressed in all tissues [42, 44]. Overexpression of *FC-II* in *Arabidopsis* reduces chlorophyll content but does not affect the expression of nuclear genes linked to photosynthesis [45]. Impaired expression of *FC-II* reduces chlorophyll content and shows a necrotic leaf phenotype [46]. The *Arabidopsis fc-II* mutant exhibits small, pale green rosette leaves, low levels of chlorophyll, carotenoid and several photosynthetic proteins, and low photosynthetic efficiency, but exhibits decreased sensitivity to salt stress [47]. These studies demonstrate that FC-II is involved in plant growth and stress responses; however, there is no report on FC-II involvement in plant immune responses.

Potato StFC-II is a chloroplast-located protein encoded by the genome DNA. In this study, we demonstrate that StFC-II is targeted by a *P. infestans* RxLR effector Pi22922 in the cytoplasm. And FC-II partially contributes to the pathogenicity of Pi22922. Overexpression of *StFC-II* enhances the host susceptibility to *P. infestans*. Pi22922 does not enter chloroplasts, but promotes StFC-II accumulation in chloroplasts accompanied by the compromised chloroplast and cytoplasm ROS burst and immune responses. This study expands our knowledge of how a chloroplast protein is hijacked by an oomycete effector to supress host resistance.

## Results

### The RxLR effector Pi22922 promotes *P. infestans* colonization

**Figure 1:**
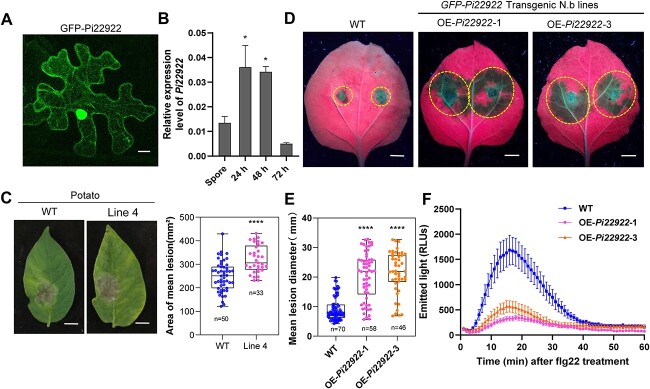
RxLR effector Pi22922 enhances *Phytophthora infestans* colonization. **A** Confocal image shows that Pi22922 localizes in the cytoplasm and nucleus. Bar, 10 μm. **B** Bar graph showing the relative expression of *Pi22922* during the *P. infestans* infection of potato leaves. *P. infestans* isolate HB09–14-2 was used for inoculation. Total RNA was isolated from the inoculated potato leaves at 24, 48, and 72 hours. *EF1α* was used as the reference gene. Error bars indicate ± SEM (one-way ANOVA, ^*^P < 0.05, three biological replicates). **C** The lesion area on the leaf of *HA-Pi22922* transgenic potato line is dramatically increased compared to the wild-type control. Data are displayed by a box-whisker plot. (*t* test, ^*^^*^^*^^*^P < 0.0001, three biological repeats). Scale bars represent 1 cm. **D** Representative images show that lesion diameter on the leaves of transgenic *N. benthamiana* lines is larger compared to the wild-type plants. Scale bars represent 1 cm. **E** Box–whisker plot shows that the lesion diameter on leaves of transgenic *Nicotiana benthamiana* is significantly bigger than that of wild-type control (one-way ANOVA, ^*^^*^^*^^*^P < 0.0001, three biological replicates). **F**. Stable expression of *Pi22922* in transgenic *N. benthamiana* leaves suppresses ROS burst. Leaves from *Pi22922*-expressing transgenic lines and WT were treated with 10 μM flg22 before ROS measurement (24 leaf discs from three plants per line). RLUs, relative luminescence units.

Due to its essential role in plant immunity, the chloroplasts are prone to be attacked by pathogen virulence factors. To investigate whether the *P. infestans* RxLR effectors manipulate the chloroplast immunity, we firstly predicted which *P. infestans* effector contains the putative chloroplast transit peptide (cTP) using LOCALIZER (https://localizer.csiro.au/) and ChloroP-1.1 (https://services.healthtech.dtu.dk/services/ChloroP-1.1/). Among them, Pi22922 is predicted to contain a putative cTP. Pi22922 encodes 488 amino acids with a signal peptide (1–23), a putative cTP (32–57), an RxLR-EER motif and a C-terminal effector domain, and does not contain other domains such as nuclear localization sequence (NLS) (Fig. S1, see online supplementary material). To confirm the subcellular localization of Pi22922 in plant cells, GFP was fused to the N-terminus of Pi22922 (without predicted signal peptide). 35S promoter-controlled *GFP-Pi22922* was transiently expressed in *Nicotiana benthamiana* leaves. GFP-Pi22922 fusion protein was stably expressed (Fig. S2A, see online supplementary material). Confocal microscopy observation showed that GFP-Pi22922 was accumulated in the cytoplasm and nucleus, but not in the chloroplast (Fig. 1A). *Pi22922* was up-regulated 24 and 48 hours after inoculation of *P. infestans* in potato leaves, indicating that Pi22922 exerts pathogenicity in the early phase of infection (Fig. 1B), and its function needs to be further investigated.

To investigate the function of Pi22922, stable overexpression (OE) *HA-Pi22922* transgenic potato line and OE *GFP-Pi22922* transgenic *N. benthamiana* lines were generated (Fig. S2B–E, see online supplementary material). *P. infestans* inoculation showed that disease lesion areas on the leaves of transgenic potato line significantly increased compared to the wild type control (Fig. 1C). Similar to the OE potato line, transgenic *N. benthamiana* lines also showed significant enhancement of *P. infestans* colonization (Fig. 1D and E). In addition, compared to wild-type leaves, earlier reactive oxygen species (ROS) burst triggerd by flg22 was inhibited in *GFP-Pi22922* transgenic *N. benthamiana* leaves (Fig. 1F). These results demonstrate that Pi22922 is a virulence factor and contributes to *P. infestans* colonization.

### Chloroplast ferrochelatase StFC-II interacts with Pi22922

A yeast-2-hybrid (Y2H) cDNA library from potato leaves infected with *P. infestans* was screened to identify putative host targets of RxLR effector Pi22922. Ten potential interacting proteins were identified (Table S1, Fig. S3A, see online supplementary material). Among them, two candidates were predicted to be the chloroplast proteins (Table S1, see online supplementary material). One protein encodes ferrochelatase II (FC-II), which is a terminal enzyme in the chloroplast heme synthesis pathway. It is hereafter called StFC-II in potato. Transient expression of *StFC-II-HA* in *N. benthamiana* significantly enhanced *P. infestans* colonization compared with EV*-HA* control (Fig. S3B, see online supplementary material). Another protein encodes peptidase M1 family protein (StPeptidase_M1). There was no significant difference in disease lesion diameter when it was transiently expressed compared to the EV*-HA* control (Fig. S3C, see online supplementary material). Thus StFC-II was selected for further study. StFC-II shares a high amino acid identity with FC-II from *Arabidopsis thaliana*, *Solanum lycopersicum* and *N. benthamiana* (Fig. S4, see online supplementary material).

**Figure 2:**
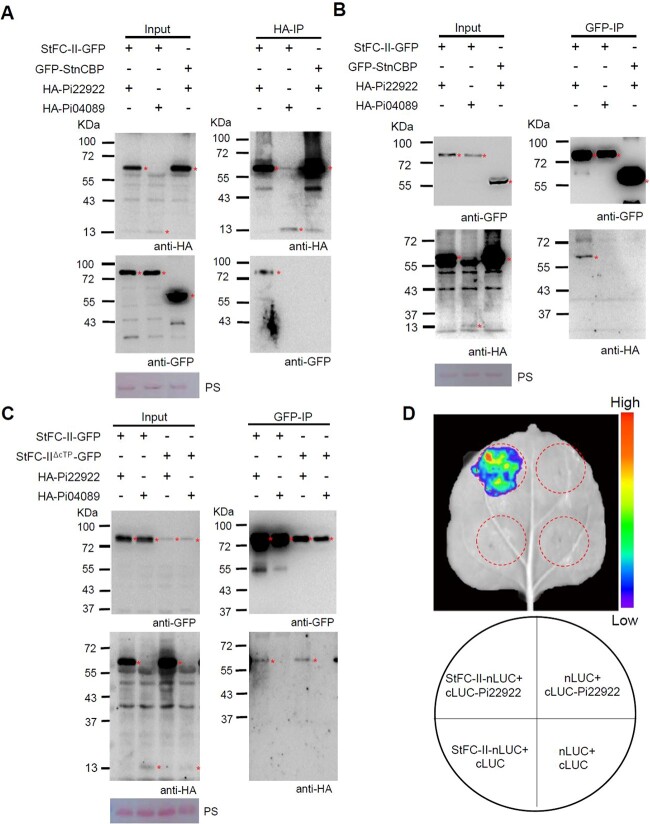
Pi22922 interacts with StFC-II *in planta*. **A** co-IP assay shows that StFC-II-GFP was specifically immunoprecipitated by HA-Pi22922, not by control HA-Pi04089. HA-Pi22922 did not immunoprecipitate the control GFP-StnCBP (a cytoplasmic protein). HA-agarose beads were used for immunoprecipitation from leaf extracts. **B** co-IP assay confirms that HA-Pi22922 was specifically immunoprecipitated by StFC-II-GFP and HA-Pi04089 did not. Control GFP-StnCBP did not immunoprecipitate HA-Pi22922. GFP-agarose beads was used for immunoprecipitation from leaf extracts. **C** co-IP assay confirms that HA-Pi22922 was immunoprecipitated by StFC-II-GFP and StFC-II^ΔcTP^-GFP (lacking chloroplast transit peptide), but control HA-Pi04089 did not. GFP-agarose beads was used for immunoprecipitation from leaf extracts. Protein expression in *Nicotiana benthamiana* leaves was represented by a ‘+’. Protein size markers were given in kDa, and protein loading was indicated by Ponceau stain (PS). ^*^ indicates target protein band. **D** Split luciferase complementation assay confirms that Pi22922 interacts with StFC-II *in planta*. The luminescence signal was detected by the plant live imager.

To confirm the interaction between Pi22922 and StFC-II, co-immunoprecipitation (co-IP) assay was performed by transient co-expression of *HA-Pi22922* or *HA-Pi04089* (another *P. infestans* effector, used as control) with *StFC-II-GFP* or *GFP-StnCBP* (cytoplasm-localized control protein) in *N. benthamiana* leaves, following immunoprecipitation with HA-agarose beads. All proteins were stably expressed. StFC-II-GFP was specifically immunoprecipitated by HA-Pi22922, not by the negative control HA-Pi04089; and HA-Pi22922 did not immunoprecipitate control GFP-StnCBP (Fig. 2A). In reverse, HA-Pi22922 was specifically immunoprecipitated by StFC-II-GFP (Fig. 2B). Chloroplast transit peptide (cTP) is an N-terminal extension that facilitates the transport and localization of cytosolically synthesized precursors into chloroplast [48, 49]. To investigate whether chloroplast transit peptide (cTP) is critical for the interaction between StFC-II and Pi22922, *StFC-II^ΔcTP^-GFP* (lacking chloroplast transit peptide) was constructed and co-IP assay was performed. Both StFC-II^ΔcTP^-GFP and StFC-II-GFP interacted with Pi22922, but they did not interact with the control Pi04089 (Fig. 2C). This result indicated that the cTP is not required for the Pi22922–StFC-II interaction. Split luciferase complementation (LUC) assay was also used to prove their interaction. Luminescence signal was only detected when *StFC-II-nLUC* and *cLUC-Pi22922* were co-expressed (Fig. 2D). StFC-II-nLUC and cLUC-Pi22922 were stable in *N. benthamiana* leaves (Fig. S5A, see online supplementary material). These results demonstrate that Pi22922 specifically targets StFC-II in plants.

### StFC-II interacts with Pi22922 in the host cytoplasm

**Figure 3:**
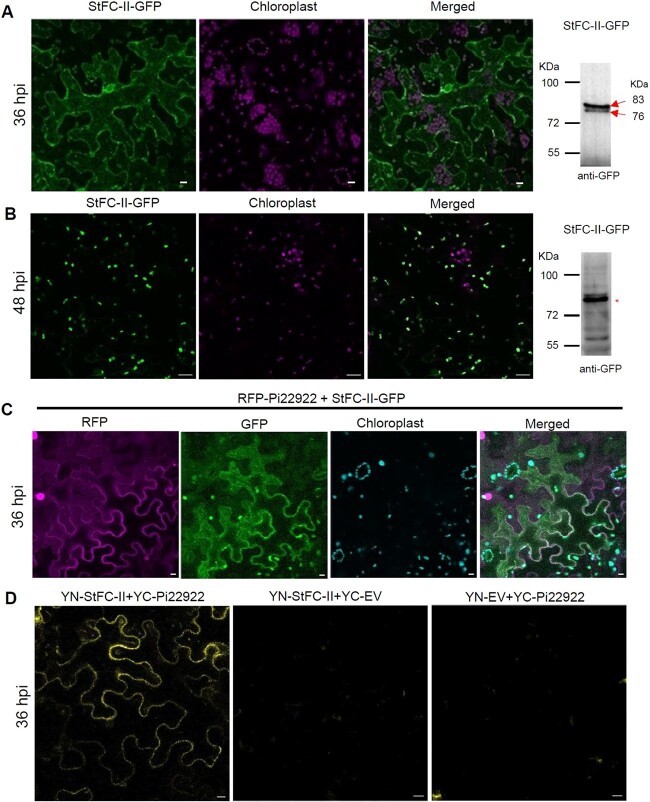
Pi22922 interacts with StFC-II in the cytoplasm. **A** Images show that StFC-II-GFP is mainly localized in the cytoplasm at 36 hpi (hours post agro-infiltration). Images are projections of confocal Z series. Fluorescence was observed on leaves 36 h after transient expression of StFC-II-GFP by agro-infiltration in *Nicotiana benthamiana* leaves. Scale bars represent 10 μm. Western blot image (on the right) shows the expression of StFC-II-GFP at 36 hpi *in planta*. Stronger precursor protein band was observed (up red arrow indicates precursor protein, down red arrow indicates mature protein). Protein size markers are indicated in kDa. **B** Confocal images showing the chloroplast localized StFC-II-GFP. Fluorescence was observed on the leaves expressing StFC-II-GFP at 48 hpi. Scale bars represent 20 μm. Right western blot image shows that the mature protein band (indicated by ^*^) is stronger than the precursor protein band at 48 hpi. **C** Images show that RFP-Pi22922 co-localizes with StFC-II-GFP in the cytoplasm at 36 hpi. Images are projections of confocal Z series. Scale bars represent 10 μm. **D** BiFC assay showing that YFP fluorescence appeared in the cytoplasm after YN-StFC-II co-expressing with YC-Pi22922 at 36 hpi. Scale bars represent 10 μm.

It has been reported that FC-II was accumulated in the chloroplast in *A. thaliana* and cucumber [40, 42–44]. GFP was fused to the C-terminus of *StFC-II* to generate *StFC-II-GFP*. Then *StFC-II-GFP* was transiently expressed in *N. benthamiana* leaves by agro-infiltration. StFC-II is a chloroplast protein encoded by nuclear DNA. Its precursor is synthesized in the cytoplasm and transported into chloroplasts led by the cTP [48, 49]. cTP is cleaved after protein is directed into chloroplasts. Confocal image showed that much StFC-II-GFP still stained in the cytoplasm at 36 hpi (hours post agro-infiltration), and the precursor protein was abundant than mature protein (Fig. 3A; Fig. S6, see online supplementary material). StFC-II-GFP was accumulated in the chloroplast at 48 hpi (Fig. 3B). Western blot indicated that StFC-II-GFP contains two bands, which represent precursor protein (large band, in the cytoplasm) and mature protein (small band, in the chloroplast), respectively. At 48 hpi, the chloroplast-located mature protein was more abundant (Fig. 3B). To verify the mature chloroplast protein band, chloroplasts were isolated and StFC-II-GFP protein in chloroplasts was detected by western blotting. The result showed that the small band is indeed the chloroplast-located mature protein (Fig. S5B, see online supplementary material). At 36 hours post agro-infiltration, StFC-II-GFP and RFP-Pi22922 were co-localized in the cytoplasm (Fig. 3C), which allows for the possibility of Pi22922 interacting with StFC-II before it is transported into the chloroplast. RFP-Pi22922 was stably expressed *in planta* (Fig. S5A, see online supplementary material). Bimolecular fluorescence complementation (BiFC) assay was performed to further verify their interaction. YFP fluorescence, produced by co-expression of YN-StFC-II and YC-Pi22922 in *N. benthamiana*, distributed only in the cytoplasm, while no fluorescence was observed in the area co-expressing YN-StFC-II with YC-EV or YN-EV with YC-Pi22922 (Fig. 3D). Taken together, results above imply that Pi22922 interacts with StFC-II in the cytoplasm.

### Overexpression of *StFC-II* enhances plant susceptibility to *P. infestans*

**Figure 4:**
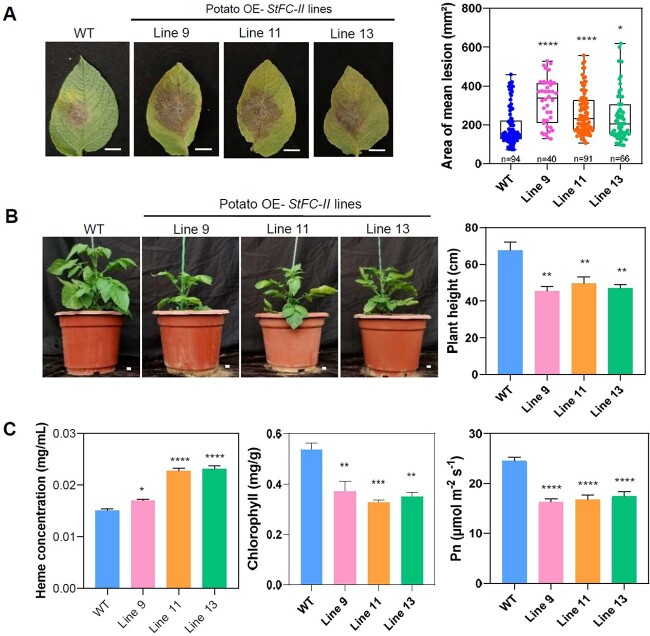
Enhanced expression of *StFC-II* increases plant susceptibility to *Phytophthora infestans*. **A** Representative leaf images and bar graph show the significantly larger lesion area in stable overexpressing *StFC-II-HA* transgenic potato lines compared with wild-type plants. Data are presented via a box–whisker plot (one-way ANOVA, ^*^P < 0.05, ^*^^*^^*^^*^P < 0.0001, four biological repeats). Scale bars represent 1 cm. **B** Overexpression of *StFC-II* reduces potato plant height. Error bars of each graph represent mean ± SEM. (one-way ANOVA, ^*^^*^P < 0.01, six plants are measured per line). **C.** Overexpression of *StFC-II* increases heme content and reduces chlorophyll content and photosynthetic efficiency. Error bars of each graph represent mean ± SEM (one-way ANOVA, ^*^P < 0.05, ^*^^*^P < 0.01, ^*^^*^^*^P < 0.001, ^*^^*^^*^^*^P < 0.0001, six plants are measured per line).

Previous research reported that FC-II produces heme for photosynthetic cytochromes, and *fc-II* mutant exhibits decreased sensitivity to salt stress in *A. thaliana* [47]. As a target of *P. infestans* effector, we speculated that StFC-II may be involved in plant immune regulation. Transgenic *N. benthamiana* plants overexpressing (OE) *StFC-II-GFP* and RNAi *NbFC-II* were generated (Fig. S7A–D, see online supplementary material). Upon *P. infestans* isolate 88069 inoculation, significantly larger lesion diameters were observed on the leaves of OE *StFC-II-GFP* transgenic *N. benthamiana* lines compared to the control plants (Fig. S7E, see online supplementary material), while smaller lesion diameters were observed on the leaves of RNAi *NbFC-II* transgenic *N. benthamiana* lines (Fig. S7F, see online supplementary material). Similar to OE *StFC-II N. benthamiana* plants, potato *StFC-II* OE transgenic lines also showed significant enhancement of *P. infestans* colonization (Fig. 4A; Fig. S7G and H, see online supplementary material). These results reveal that enhanced expression of *StFC-II* increases plant susceptibility.

As reported previously, significantly impaired expression of *FC-II* led to stunted plants, reduced chlorophyll content, leaf necrosis, and decreased photosynthetic rate [46, 47]. The plant growth and development of three RNAi *NbFC-II* transgenic *N. benthamiana* lines with relatively low gene silencing efficiency were less affected (Fig. S7C and D, see online supplementary material). However, transgenic potato lines overexpressing (OE) *StFC-II-HA* showed reduced plant height (Fig. 4B). The heme content in leaves of *StFC-II-HA* OE potato lines was significantly higher than that of wild-type plants, while the chlorophyll content and photosynthetic rate were significantly lower than that of wild-type plants (Fig. 4C), demonstrating that overexpression of *StFC-II-HA* in chloroplasts disturbs the balance of heme and chlorophyll biosynthesis and decreases photosynthetic rate.

### Chloroplast localization of StFC-II is essential for promoting susceptibility

**Figure 5:**
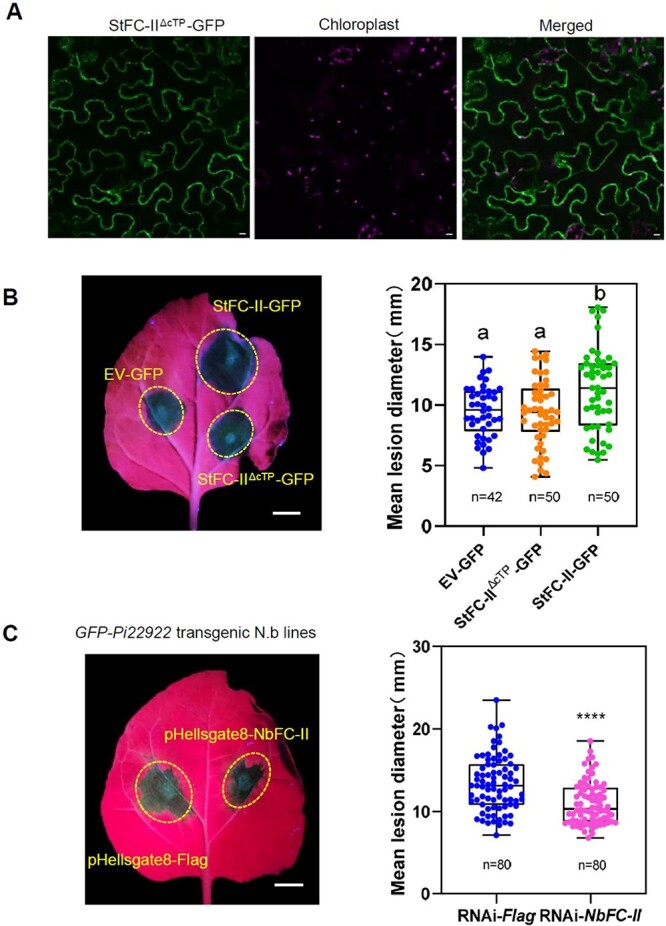
Chloroplast localization of StFC-II is essential for it to promote susceptibility. **A** Confocal images show that StFC-II^ΔcTP^-GFP (lacking chloroplast transit peptide) is localized in the cytoplasm. Image sets are single optical sections. Photo taken at 48 h after ago-infiltration. Scale bars represent 10 μm. **B** Representative leaf image and bar graph showing disease lesion diameter on the leaf transiently expressing *StFC-II^ΔcTP^-GFP*, EV*-GFP*, or *StFC-II-GFP* (one-way ANOVA, three biological replicates). **C** Box–whisker plot showing *in situ* transiently silencing *NbFC-II* impairs the ability of Pi22922 to promote host susceptibility. *In situ* transiently silencing *NbFC-II* was conducted by agro-infiltrating RNAi vector pHellsgate8-*NbFC-II* in *Nicotiana benthamiana* leaves. *Phytophthora infestans* isolate 88069 inoculation was performed 24 hours after agro-infiltration. Disease lesion diameters were measured 6–7 days after inoculation. Data are presented via a box–whisker plot (*t* test, ^*^^*^^*^^*^P < 0.0001, four biological repeats). Scale bars represent 1 cm.

FC-II is synthesized in the cytoplasm and transported into chloroplasts. To investigate whether chloroplast localization is critical for StFC-II to promote susceptibility, *StFC-II^ΔcTP^-GFP* (lacking chloroplast transit peptide) was transiently expressed in *N. benthamiana*. StFC-II^ΔcTP^-GFP was observed to be localized in the cytoplasm with laser confocal microscopy, but not in the chloroplast (Fig. 5A). This result demonstrated that cTP is required for the chloroplast localization of StFC-II. To test its function, *StFC-II^ΔcTP^-GFP*, EV*-GFP*, and *StFC-II-GFP* were transiently expressed in different areas on the same *N. benthamiana* leaf for 24 hours, and then inoculated with *P. infestans* isolate. Results showed that there was no significant difference in lesion diameter in the area transiently expressing *StFC-II^ΔcTP^-GFP* or EV*-GFP* control, while disease lesion diameter in the area transiently expressing *StFC-II-GFP* was significantly larger than in the *StFC-II^ΔcTP^-GFP* and EV*-GFP* infiltrated areas (Fig. 5B). StFC-II^ΔcTP^-GFP was normally expressed *in planta* (Fig. S5A, see online supplementary material). These results implied that StFC-II promotes susceptibility relying on its chloroplast localization.

As shown above, both Pi22922 and StFC-II promote *P. infestans* colonization, and Pi22922 interacts with StFC-II *in planta*. We wonder whether StFC-II is relied upon by *P. infestans* effector Pi22922 to promote host susceptibility. The *in situ* RNAi method was performed to transiently silence *NbFC-II* in *GFP-Pi22922* transgenic *N. benthamiana* leaves. *NbFC-II* expression level was significantly reduced in transiently silencing sites (Fig. S8, see online supplementary material). Subsequent *P. infestans* inoculation showed that transiently silencing *NbFC-II* impaired the ability of Pi22922 to promote host susceptibility compared to the control vector pHellsgate8-*Flag* (Fig. 5C).

### Overexpression of *StFC-II* compromises plant immune responses

**Figure 6:**
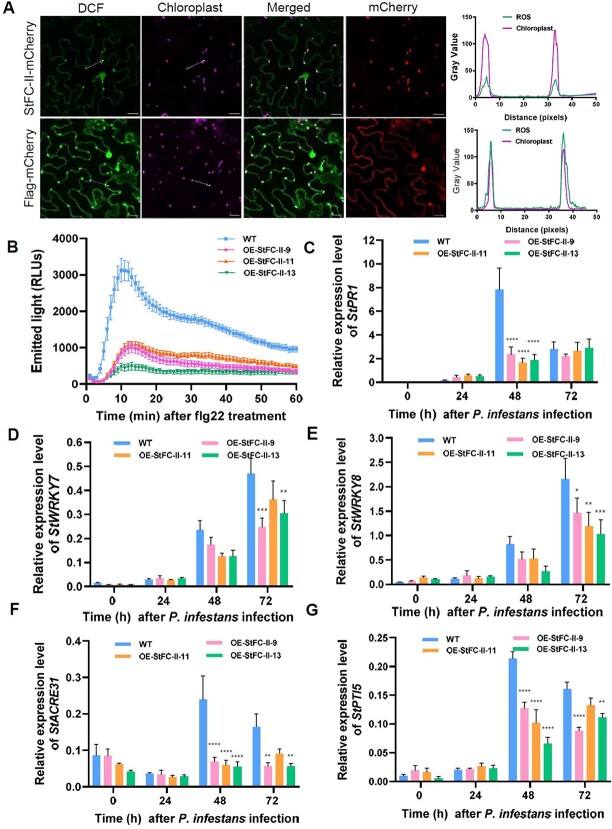
Overexpression of *StFC-II* compromises both the chloroplast and cytoplasm ROS accumulation and immune responses. **A** Confocal images show that transient expression of *StFC-II-mCherry* results in reduced ROS accumulation in chloroplasts compared with that of control *Flag-mCherry*. Forty-eight hours after agro-infiltration, leaves were infiltrated with DCF-DA and placed in the dark for 1 h and then induced by flg22 for 10 minutes. Green fluorescence indicates ROS signal. Scale bars represent 20 μm. Right plots showing the green fluorescence intensity of two chloroplasts compared with chloroplast autofluorescence (pink). **B** StFC-II suppresses H_2_O_2_ production in *StFC-II* overexpression (OE) transgenic potato leaves. Leaves from *StFC-II* OE transgenic potato lines and WT plants were induced by 10 μM flg22. RLUs, relative luminescence units. **C**–**G**. Expression levels of *StPR1*, *StWRKY7*, *StWRKY8*, *StACRE31*, and *StPTI5* in *StFC-II* OE transgenic and WT plants. Detached leaves were inoculated by *Phytophthora infestans* sporangia. Total RNA was extracted from the leaf samples collected at 0, 24, 48, and 72 h post inoculation. Gene expression levels were measured by qRT-PCR. *StEF1α* is used as the plant reference gene. Error bars indicate ± SEM of three biological replicates (one-way ANOVA, ^*^P < 0.05, ^*^^*^P < 0.01, ^*^^*^^*^P < 0.001, ^*^^*^^*^^*^P < 0.0001).

Overexpression of *StFC-II* promotes plant susceptibility to *P. infestans*, and that depends on its chloroplast localization (Figs 4Aand 5). The accumulation of reactive oxygen species (ROS) is the key defense signal of the chloroplast. To investigate whether excessive StFC-II disturbs ROS accumulation in the chloroplast (cROS), we transiently expressed *StFC-II-mCherry* and *Flag-mCherry* in *N. benthamiana* leaves and then monitored H_2_O_2_ accumulation using a fluorescent indicator of ROS, 2′ 7′-dichlorodihydrofluorescein (DCF-DA). Green fluorescence indicates ROS signal. The fluorescence signal in chloroplasts on the leaves expressing *StFC-II-mCherry* was observed to be very weak compared with that on the control leaves after flg22 treatment using laser confocal microscopy (Fig. 6A), indicating ROS production was suppressed in chloroplasts. In addition, H_2_O_2_ production was measured in leaves of *StFC-II* OE transgenic potato lines and WT plants responding to the flg22 treatment. Obviously, H_2_O_2_ production was suppressed in the *StFC-II* OE transgenic potato leaves compared to the wild-type control (Fig. 6B). Therefore, the expression of *StFC-II* compromises ROS accumulation both in the chloroplast and cytoplasm. We also found that ROS burst was suppressed in *Pi22922* stable transgenic *N. benthamiana* leaves in response to flg22 treatment (Fig. 1F). Thus, we speculated that Pi22922 inhibits H_2_O_2_ accumulation and that may partially depend on StFC-II.

To further examine the effects of StFC-II on plant immunity, the expression levels of PTI marker genes *StPR1*, *StWRKY7*, *StWRKY8*, *StACRE31*, and *StPTI5* were tested by qRT-PCR in *StFC-II*-expressing transgenic potato lines and WT plants during the *P. infestans* infection stages. Compared to the wild-type plants, the expression levels of PTI marker genes were significantly suppressed in the transgenic lines in certain stages (Fig. 6C–G). Collectively, the above results revealed that overexpression of *StFC-II* in plants compromises both the chloroplast and cytoplasm ROS accumulation and PTI immune responses.

### Pi22922 facilitates accumulation of StFC-II in the chloroplasts and enhances its stability

**Figure 7:**
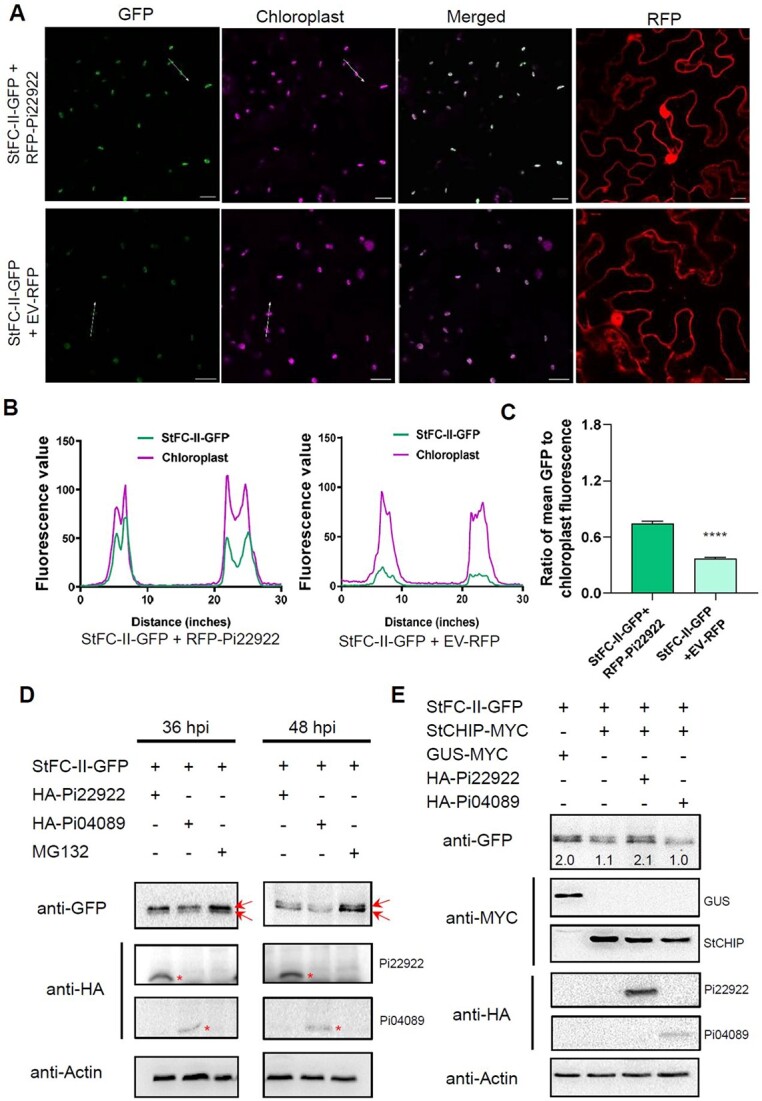
Pi22922 facilitates entry of StFC-II into chloroplasts and stabilizes it. **A** Confocal images showing stronger green fluorescence signal in the chloroplast in the area co-expressing *StFC-II-GFP* with *RFP-Pi22922* rather than that in the control infiltrated areas. 48 hours after agro-infiltration. Bar, 20 μm. **B** Plots showing the fluorescence intensity values of two chloroplasts marked by white arrows in the area co-expressing *StFC-II-GFP* with *RFP-Pi22922* or *StFC-II-GFP* with control EV-*RFP*. Chloroplast autofluorescence is indicated by pink color. **C** Graph shows that the ratio of green fluorescence to chloroplast autofluorescence is significantly higher in the leaf area co-expressing *StFC-II-GFP* with *RFP-Pi22922* compared to that in the control leaf area co-expressing *StFC-II-GFP* with EV-*RFP*. The fluorescence intensity of 100 chloroplasts in different areas was measured. Error bars show ± SEM (*t* test, ^*^^*^^*^^*^P < 0.0001). **D** Western blot shows that HA-Pi22922 stabilizes StFC-II-GFP, but control HA-Pi04089 does not (up arrow indicates precursor protein band, down arrow indicates mature protein band) at 36 and 48 hours post agro-infiltration. The protein level of StFC-II increased greatly in the presence of MG132. MG132 was produced by a construct via Agro*-*infiltration. Agrobacteria concentration (OD_600_ value) was adjusted to 0.3 for expressing MG132. **E** Pi22922 stabilizes StFC-II by inhibiting E3 ubiquitin ligase StCHIP-mediated degradation. Western blot shows that Pi22922 stabilizes StFC-II by inhibiting StCHIP-mediated degradation, but control Pi04089 does not. Constructs expression in *Nicotiana benthamiana* leaves is represented by a ‘+’. Protein size markers are indicated in kDa, and the same protein loading is indicated by Actin amounts using Actin antibody.

Pi22922 interacts with StFC-II in the cytoplasm (Fig. 3C and D); however, StFC-II enhances plant susceptibility in the chloroplast (Fig. 5). To investigate how Pi22922 affects the function of StFC-II, *StFC-II-GFP* and *RFP-Pi22922* were transiently co-expressed in *N. benthamiana* leaves. We observed that the green fluorescence signal of chloroplasts in the area co-expressing *StFC-II-GFP* and *RFP-Pi22922* was stronger than that in the control area (Fig. 7A and B). The fluorescence intensity of about 100 chloroplasts in different areas was measured. The ratio of green fluorescence to chloroplast autofluorescence was higher in the areas co-expressing *StFC-II-GFP* with *RFP-Pi22922* compared to co-expressing *StFC-II-GFP* with control EV*-RFP* (Fig. 7C), indicating the presence of Pi22922 enhances StFC-II-GFP accumulation in chloroplasts. The protein abundance of StFC-II was tested in conditions of with or without Pi22922 by west blotting. Constructs combination of StFC-II-GFP with HA-Pi22922 or StFC-II-GFP with HA-Pi04089 were transiently co-expressed in *N. benthamiana* leaves. In the presence of HA-Pi22922, the abundance of StFC-II-GFP precursor protein was much higher than in the negative control at 36 hours post agro-infiltration (hpi) (Fig. 7D). The protein abundance of mature protein was also increased in the presence of HA-Pi22922 compared to the condition with HA-Pi04089 at 48 hpi (Fig. 7D). Upon MG132 treatment, the abundance of both precursor and mature protein increased greatly (Fig. 7D). These results revealed that Pi22922 stabilizes StFC-II in the cytoplasm and facilitates its accumulation in chloroplasts *in planta*.

Heat Shock Protein Cognate 70-4 (Hsc70-4) and E3 ubiquitin ligase, CHIP, were shown to mediate chloroplast precursor proteins degradation through the ubiquitin-26S proteasome system in the cytoplasm [50, 51]. To determine whether Pi22922 could prevent StFC-II degradation in the cytoplasm through this mechanism, we co-expressed *Pi22922* with *StFC-II*, *StCHIP* and tested the protein level of StFC-II. The protein level of StFC-II was lower when StFC-II was co-expressed with StCHIP compared to when it was co-expressed with control GUS. However, in the presence of Pi22922, StFC-II was stabilized and the protein level was much higher than in the presence of control Pi04089 (Fig. 7E; Fig. S10, see online supplementary material), demonstrating that Pi22922 inhibits the degradation of StFC-II mediated by E3 ubiquitin ligase StCHIP in the cytoplasm.

Taken together, the above results revealed that Pi22922 stabilizes StFC-II in the cytoplasm and facilitates its accumulation in chloroplasts.

## Discussion

Oomycete RxLR effectors are critical weapons for their virulence. Understanding the molecular dialogues between RxLR effectors and hosts is crucial for effective disease control. Chloroplast plays a central role in plant photosynthesis and contributes to immunity. Diverse chloroplast proteins were hijacked by pathogen effectors to facilitate their infection and proliferation [31, 32]. In this study, we found that a chloroplast protein StFC-II was targeted by RxLR effector Pi22922 (Fig. 2). FC-II is the terminal enzyme of the heme biosynthetic pathway. In addition to producing heme for photosynthetic cytochromes, FC-II also produces heme for stress responses. In *A. thaliana*, *fc2-1* knock-down plants showed altered photosynthetic activity with abnormal growth and development. Furthermore, impairment of *FC-II* led to reduced sensitivity to salt and biotic stress [47]. We demonstrate that overexpression of *StFC-II* enhanced potato and *N. benthamiana* plants’ susceptibility to *P. infestans* while silencing *NbFC-II* in *N. benthamiana* inhibited the *P. infestans* colonization (Fig. 4; Fig. S7, see online supplementary material). We also found that the pathogenicity of Pi22922 partially relayed on the FC-II (Fig. 5C). Overexpression of *StFC-II* in potatoes inhibited both the chloroplast and cytoplasm ROS (cROS) accumulation and reduced the transcript levels of PTI marker genes (Fig. 6), which indicated decreased immunity. Thus, StFC-II could be regarded as a susceptibility factor (S factor). Our results firstly demonstrate that, besides responding to abiotic stresses, FC-II is also involved in plant immune responses.

Heme biosynthesis shares the same upstream pathway with chlorophyll biosynthesis in plant plastids. FC-II is the last enzyme of heme-producing branch biosynthetic pathway. Expression alteration of *FC-II* may disrupt the subtle balance of chlorophyll and heme biosynthesis, which will lead to abnormal chloroplast metabolism, plant growth, and development, resulting in damage to the whole plant. Impaired expression of *FC-II* in *Arabidopsis* leads to stunted plants, reduced chlorophyll content, leaf necrosis, and decreased photosynthetic rate [46, 47]. The leaves of *N. bentamiana FC-II* RNAi lines turned pale green in the later stage (Fig. S9, see online supplementary material), which is similar to the *Arabidopsis fc-II* mutants [47]. However, we found that transgenic potato lines overexpressing *StFC-II-HA* showed increased heme content, reduced chlorophyll content, decreased photosynthetic rate, and plant height (Fig. 4B and C), indicating that overexpressing *StFC-II* disrupted the balance of chlorophyll and heme biosynthesis, which leads to the abnormal plant growth.

It is well known that excessive ROS accumulation could cause plant cell death at the infection site to hinder the nutrient uptake for the growth and development of the biotrophic and semi-biotrophic pathogens [52]. Previous research has shown that pathogen effectors target different photosynthesis components to reduce photosynthetic efficiency, ROS accumulation, and plant basal defense. *P. syringae* effector HopN1 interactes with PsbQ, a member of the oxygen-evolving complex of photosystem II (PSII), and cleaves it, thereby reducing cROS generation, callose formation and inhibiting cell death [36]. Wheat stripe rust fungus effector Pst_12806 interacts with the wheat TaISP protein, a putative component of the cytochrome b6-f complex, to attenuate photosynthetic rate, decrease ROS accumulation and inhibit plant defenses [38]. PsbP (oxygen-evolving enhancer 2) was targeted and stabilized by *P. viticola* effector RxLR31154, thereby inhibiting ROS production in grapevine [39]. We revealed that overexpression of *StFC-II* repressed both the cROS and cytoplasm ROS accumulation in potato and *N. benthamiana* (Fig. 6A and B). We supposed that the balance disruption of chlorophyll and heme biosynthesis affects photosynthesis. Photosynthesis could provide carbon skeleton, energy, reducing power for the synthesis of defense-related signaling molecules or their precursors such as SA, ABA, ET, JA precursor, ROS and^**.**^NO. Reduced photosynthetic rate in *StFC-II* OE transgenic plants may weaken the cROS production and synthesis of defense-related signaling molecules, that in turn attenuates cytoplasm and nuclear immune responses.

**Figure 8:**
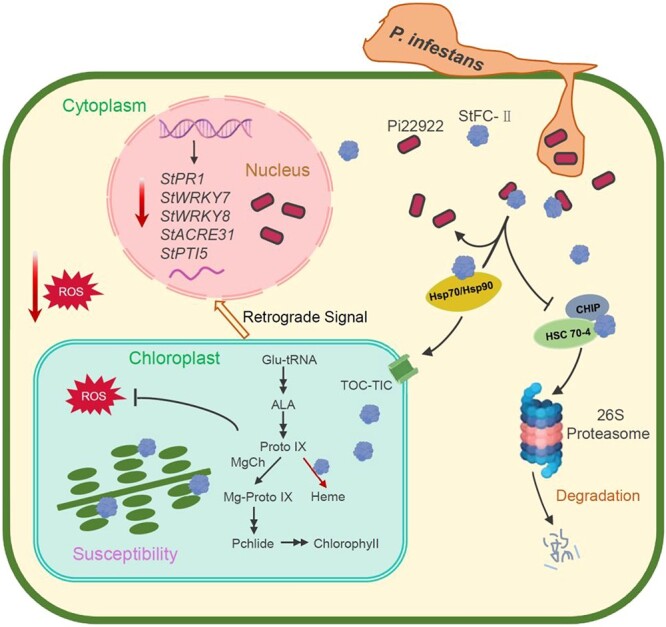
A proposed model of how StFC-II was manipulated by RxLR effector Pi22922 to suppress host imunitiy. *Phytophthora infestans* RxLR effector Pi22922 was secreted from haustoria and translocated in the nucleus and cytoplasm. In cytoplasm, Pi22922 targeted the chloroplast protein StFC-II and inhibited its degradation mediated by E3 ubiquitin ligase CHIP, thereby promoting its accumulation in chloroplasts. Much StFC-II accumulation in the chloroplast disrupts the subtle balance of chlorophyll and heme biosynthesis, which leads to decreased cROS and cytoplasm ROS production and inhibition of the defense-related genes’ expression. Pi22922 may also target host nuclear-located proteins to simultaneously suppress immunity.

Pathogen effectors disturb the chloroplast immunity by different mechanisms of action [31, 32]. Disturbing the nuclear-coded chloroplast proteins translocation is one of tactics. Effectors Pst_4 and Pst_5 suppress plant defenses in the cytoplasm by preventing TaISP from entering chloroplasts, thereby inhibiting host ROS accumulation and enhancing fungal infection [33]. Our results showed that effector Pi22922 localized both in the nuclear and cytoplasm (Fig. 1A). StFC-II is a nuclear-coded chloroplast protein. We found that StFC-II-GFP was localized both in the cytoplasm and chloroplasts 36 h post transient expression in *N. benthamiana* leaves and it was almost transferred into the chloroplasts 48 h post agro-infiltration (Fig. 3). However, Pi22922 interacts with StFC-II in the cytoplasm (Fig. 3C and D). When Pi22922 was co-expressed with StFC-II-GFP, more StFC-II-GFP accumulated in chloroplasts (Fig. 7). It is worth noting that chloroplast localization of StFC-II is essential for it to promote plant susceptibility (Fig. 5A and B). Given that they co-localize in the cytoplasm and Pi22922 interacts with both StFC-II^ΔcTP^-GFP and StFC-II-GFP (Fig. 2C), the explanation is that Pi22922 stabilizes StFC-II in the cytoplasm, leading to more StFC-II being localized and accumulated in chloroplasts, which in turn disturbs the chloroplast-mediated immunity (Fig. 8). In this study, we noticed that pathogenicity of Pi22922 partially relays on the FC-II function (Fig. 5) and its protein nuclear distribution, indicating Pi22922 may target other plant nuclear-located proteins to simultaneously facilitate its virulent function, which requires further exploration.

Chloroplast is a crucial plant organelle for carbon fixation, ATP, and NADPH production, and the biosynthesis of many crucial organic molecules that are essential for plant growth and defense. Our results showed that Pi22922 stabilizes StFC-II in the cytoplasm and facilitates its accumulation in chloroplasts (Fig. 7). Pi22922 inhibits the degradation of StFC-II mediated by E3 ubiquitin ligase StCHIP (Fig. 7E; Fig. S10, see online supplementary material). The detailed mechanism of whether StFC-II is directly targeted and ubiquitinated by E3 ubiquitin ligase StCHIP and whether Pi22922 inhibits the degradation of StFC-II by weakening the interaction between StFC-II and StCHIP requires further investigation. Moreover, the mechanism of why more StFC-II accumulation in the chloroplasts inhibits plant defense responses remains to be determined. Nevertheless, StFC-II could be a potentially important target to be protected, avoiding disturbing by effectors using gene editing technology, to improve host disease resistance without affecting normal biological functions.

## Materials and methods

### Plant material and growth condition

*Nicotiana benthamiana* plants were grown in a growth chamber at 22°C for 16 hours of light and 18°C for 8 hours at night. Experiments required approximately 4-week-old *N. benthamiana* plants. Potato variety “E-potato-3” (E3) was used for transformation. Potato plants were grown in a greenhouse with natural conditions.

### Plasmid construction

*Pi22922* gene fragment without the signal peptide was amplified from genomic DNA of *P. infestans* isolate T30–4. To create entry clone, *Pi22922* was amplified using gene-specific primers with attB recombination sites, and recombined into pDONR201 (INVITROGEN, Carlsbad, California, USA). The entry clone was recombined into destination vectors, including pK7WGR2 and pDEST32. *Pi22922* was cloned with primers containing the restriction enzyme sites and constructed into the following vectors: pH 7-LIC7.0-N-GFP, pH 7-LIC7.0-N-3 × HA, p1300-35S-cLUC. The full-length of *StFC-II* gene was cloned from potato cDNA and cloned into pH 7-LIC7.0-C-GFP, pH 7-LIC7.0-C-3 × HA, p1300-35S-nLUC and p1300-35S-mCherry. StFC-II^ΔcTP^-GFP was generated by the Gateway® (INVITROGEN, Carlsbad, California, USA) method. Specific fragment of *NbFC-II* was cloned into the RNAi vector pHellsgate 8 for *NbFC-II* silencing. For bimolecular fluorescence complementation (BiFC) assay, the N-terminal (YN) and C-terminal (YC) fragments of YFP were fused to StFC-II and Pi22922, respectively. All primers are listed in Table S2 (see online supplementary material).

### Yeast-two-hybrid

For Y2H screen, pDEST32-Pi22922 was used as the bait and performed according to the previous method [25].

### *Agrobacterium*-mediated transient expression

Constructs were electroporated into *Agrobacterium tumefaciens* GV3101. After being cultivated for a full night in yeast-extract and beef (YEB) medium, *A. tumefaciens* were centrifuged and resuspended in MMA buffer containing 10 mM MES, 10 mM MgCl_2_, and 200 mM acetosyringone. For confocal imaging, OD_600_ value was adjusted to 0.05–0.1. For Western blot analysis, OD_600_ value was 0.5. For agro-infiltration and infection experiments, concentrations were adjusted to 0.1. For expressing MG132, OD_600_ value was 0.3. *A. tumefaciens* suspension was incubated in the dark at room temperature for at least two hours before infiltrating *N. benthamiana* leaves.

### Potato and *N. benthamiana* transformation

Micro tuber discs were used as explants, *A. tumefaciens* GV3101 harboring StFC-II-HA vector was transformed into the potato cultivar E3 according to Guo *et al.* [53]. PCR and semi-quantitative RT-PCR were used to examine the putative transgenic plants. For semi-quantitative RT-PCR, *StEF1α* was utilized as the reference gene (primers are listed in Table S2, see online supplementary material).

*A. tumefaciens* containing GFP-Pi22922, StFC-II-GFP, or pHellsgate8-NbFC-II vector were transformed into leaf discs of *N. benthamiana* according to Zhou et al. [26]. The rooting screening medium contained kanamycin (50 mg/L). Gene expression of positive lines was tested by RT-PCR and Western blot (primers are shown in Table S2, see online supplementary material).

### *P. infestans* inoculation assay

*N. benthamiana* leaves were infected with *P. infestans* isolate 88069, while potato leaves were infected with isolate HB09-14-2. After two to three weeks of culture on rye agar medium at 20°C, sporangia were collected and suspended in ddH_2_O. To inoculate *N. benthamiana*, isolate 88069's sporangia concentration was adjusted to 1.5 × 10^5^ /mL, whereas isolate HB09-14-2's sporangia concentration was 0.9 × 10^5^/mL for inoculating potato leaves. 10 μL droplets were inoculated onto detached *N. benthamiana* leaves (two sites per leaf) or potato leaves (one site per leaf). Lesion sizes were measured at 5–7 days after inoculation.

### Chloroplast isolation

Chloroplasts were extracted from *N. benthamiana* leaves using Minute™ Chloroplast Isolation Kit (INVENT, Beijing, CN).

### Western blot

Leaf samples were quickly frozen using liquid nitrogen, then ground into powder. Protein extraction buffer (10% glycerol, 25 mM Tris–HCl pH 7.5, 1 mM EDTA, 150 mM NaCl) with 10 mM DTT, protease inhibitor cocktail, 1 mM phenylmethylsulfonyl fluoride (PMSF), and 0.2% NP-40 was added to sample powder. Proteins of transgenic lines were extracted using RIPA buffer with protease inhibitor cocktail, and 1 mM phenylmethyl sulfonylfluoride (PMSF). Samples were vortexed, then put on ice before centrifuge at 4°C. The supernatants were boiled for ten minutes at 95°C and then mixed with SDS sample buffer. Proteins were separated on 6–10% SDS–PAGE gels and transfered onto polyvinylidene fluoride (PVDF) membrane. Protein bands were visible following incubation with matching antibodies using chemiluminescent ECL (Servicebio, Wuhan, China).

### Co-immunoprecipitation assay

Proteins were extracted in 800 μL protein extraction buffer. Samples were shaken and put on ice for 30 minutes before being centrifuged at 4°C until they were clear. A 50 μL input sample was removed from the supernatant and mixed with 2 × SDS sample buffer and heated at 95°C for 10 min. A total of 750 μL supernatant was incubated with agarose beads for 2 h at 4°C. Then agarose beads were washed with 500 μL ice-cold wash buffer (10% glycerol, 25 mM Tris–HCl pH 7.5, 1 mM EDTA, 150 mM NaCl, protease inhibitor cocktail, 1 mM PMSF) four times. After that, the agarose beads were resuspended in 2 × SDS sample buffer and boiled for 10 minutes at 95°C. Immunoprecipitated proteins were tested by Western blot as previously described processing.

### qRT-PCR

RNA was extracted using Total RNApure Reagent (ZOMANBIO, Wuhan, Hubei, CN). Reverse transcription was performed with All-In-One 5 × RT MasterMix (ABM, Vancouver, British Columbia, CAN) using 1 μg of total RNA. Quantitative PCR (qPCR) was performed with BlasTaq 2 × qPCR MasterMix (ABM, Vancouver, British Columbia, CAN) on an ABI7300 PCR machine (ABI, Norwalk, Connecticut, USA). *PiActin* was used as the reference gene of *P. infestans*. *NbEF1α* and *StEF1α* were used as the reference genes of *N. benthamiana* and potato. qPCR primers ware listed in Table S2 (see online supplementary material).

### Confocal imaging

*N. benthamiana* leaves producing fluorescent fusion proteins were observed using a Leica LCS confocal microscope. GFP images were taken between 498 and 534 nm of emission and 488 nm of laser excitation. At 600 nm to 630 nm emission and 561 nm laser excitation, RFP and mCherry images were recorded. Chloroplast autofluorescence was recorded between 650 and 690 nm in emission and 630 nm in laser excitation. Split-YFP images were obtained between 530 and 575 nm of emission and 514 nm of laser excitation.

### Split luciferase complementation assay

The spilt-LUC vector pCAMBIA-1300 was used for construction. nLUC and cLUC are control vectors, and they contain nLUC and cLUC coding sequences with initiation and stop codon. *A. tumefaciens* containing corresponding constructs were centrifuged and resuspended in MMA buffer. The concentration of OD_600_ value was adjusted to 0.5. After being incubated for two hours, suspensions were infiltrated into *N. benthamiana* leaves. Two days after infiltration, 15 mM of luciferin was sprayed onto the infiltrated sites of detached leaves. Fluorescence on leaves was observed 10 min after luciferin treatment. The images were captured using LB985 NightSHADE (Germany).

### ROS detection assay

The luminescence intensity was detected by a microplate reader(TECAN, Männedorf, Zürich, CH) and the level of ROS production was calculated. The luminol chemiluminescence assay was used to monitor ROS production in leaves after flg22 treatment. Four-week-old plants were sampled, and their leaves were cut into disks with a diameter of 3 mm. The disks were then pre-incubated for 12 hours in the dark in 200 μL of sterile distilled water. After removing sterile distilled water, 100 μL reaction solution of 1 μM flg22, 0.2 mg/mL HRP and 1 mM luminol was added. Samples were quickly placed into a microplate reader, and luminescence intensity was detected within 80 minutes. The analysis of ROS data was performed as described previously [54].

2′ 7′-dichlorodihydrofluorescein (DCF-DA) was used to detect the production level of ROS in chloroplasts. DCF oxidation generates green fluorescence in chloroplasts. Following a 48-hour transient expression of StFC-II-mCherry and Flag-mCherry in *N. benthamiana*, the leaves were infiltrated with 10 μM DCF and exposed to darkness for one hour. Then, leaves were treated with 10 μM flg22. A confocal microscope was used to detect the intensity of the fluorescence. ImageJ was used to measure the intensity of the fluorescence.

### Chlorophyll content and photosynthesis measurement

Chlorophyll content was measured using 0.1 g fresh leaf sample. Chlorophyll was extracted using 2 mL ethanol under dark conditions. The absorbance at wavelengths 665 nm (D_665_) and 649 nm (D_649_) was measured on an Infinite M200 microplate reader (TECAN, Männedorf, Zürich, CH). Chlorophyll content was calculated according to the formula. Chlorophyll concentration (C) = 18.08 D_649_ + 6.63 D_665_, unit is mg/L. Chlorophyll content (mg/g) = C × V/A/1000. C is chlorophyll concentration (mg/L), V is the total volume of the extract (ml), and A is the fresh weight of the leaves (g).

Four-week-old potato plants were used for net photosynthetic rate (Pn) measurement. Pn was measured from 9:00 to 11:00 a.m. on a sunny day. Six leaves from each line were selected for measurement with a portable photosynthesis system (LI-COR, Lincoln, Nebraska, USA).

### Heme content measurement

Heme content was measured using 3 g leaf sample. The sample was homogenized with 10 mL acetone/ ddH_2_O (99: 1, v/v) and (4: 1, v/v) until a colorless product was obtained. After centrifugation at 4000 *g* for 20 min at 4°C, the supernatant was discarded. The pellet was suspended twice in 5 mL HCl/acetone/ddH_2_O (5: 80: 15, v/v/v). The resultant supernatants were combined and extracted with ether. The heme was dried using nitrogen blowing. After adding 7.44 mL ddH_2_O and 0.48 mL of 5 N NaOH to dissolve the heme, 12 mL ddH_2_O and 4.08 mL pyridine was used to fix the volume. After dropping potassium persulfate and potassium ferricyanide, the absorbance at wavelengths 574.5 nm (D_574.5_) was measured on an Infinite M200 microplate reader (TECAN, Männedorf, Zürich, CH). Heme content was calculated according to the formula. Heme dilution multiple (C) = (D_574.5_–0.07506221)/2.6814496. Heme concentration (Ci) (mg/mL) = 0.5C. C is heme dilution multiple, Ci is heme concentration (mg/mL).

### Statistics methods

GraphPad Prism 8.0 software (GraphPad Prism Software Inc.) was used to perform pairwise or multiple comparisons and one-way ANOVA on all data and statistic analysis. All values and error bars presented are means ±SEM of three or more experimental replicates.

## Supplementary Material

Web_Material_uhae149

## Data Availability

The data underlying this article are available in the article and in its online supplementary data.
